# Supplementary material for: Hippocampal subfield volumes and pre-clinical Alzheimer’s disease in 408 cognitively normal adults born in 1946
Source: PLoS One. 2019 Oct 17;14(10):e0224030. doi: 10.1371/journal.pone.0224030 (PMC6797197; doi:10.1371/journal.pone.0224030)
Supplement: Attachment — Submitted filename: 1946hsf_rebuttal_jms.docx [file pone.0224030.s001.docx]

**RESPONSE TO COMMENTS IN BOLD AND RED FONT**

**From:** "PLOS ONE" <[em@editorialmanager.com](mailto:em@editorialmanager.com)>
**Date:** 22 August 2019 at 10:57:10 BST
**To:** "Jonathan Schott" <[j.schott@ucl.ac.uk](mailto:j.schott@ucl.ac.uk)>
**Subject:** **PLOS ONE Decision: Revision required [PONE-D-19-21691] - [EMID:f55a8d4587c52867]**
**Reply-To:** "PLOS ONE" <[plosone@plos.org](mailto:plosone@plos.org)>

CC: [yuankai.huo@vanderbilt.edu](mailto:yuankai.huo@vanderbilt.edu)

PONE-D-19-21691
Hippocampal subfield volumes and pre-clinical Alzheimer’s disease in 408 cognitively normal adults born in 1946
PLOS ONE

Dear Dr Schott,

Thank you for submitting your manuscript to Plos One. Your paper has been reviewed by experts in the field. The reviews have been considered by the Handling Editor and the editorial team. Based on the comments of these reviewers, we feel a major revision would be appropriate. 

Therefore, we invite you to submit a revised version of the manuscript that addresses the points raised during the review process. Please make sure to address ALL comments from the reviewers in a point-to-point manner.

We would appreciate receiving your revised manuscript by Oct 06 2019 11:59PM. When you are ready to submit your revision, log on to <https://www.editorialmanager.com/pone/> and select the 'Submissions Needing Revision' folder to locate your manuscript file.

If you would like to make changes to your financial disclosure, please include your updated statement in your cover letter.

To enhance the reproducibility of your results, we recommend that if applicable you deposit your laboratory protocols in [protocols.io](http://protocols.io), where a protocol can be assigned its own identifier (DOI) such that it can be cited independently in the future. For instructions see: [http://journals.plos.org/plosone/s/submission-guidelines#loc-laboratory-protocols](https://eur01.safelinks.protection.outlook.com/?url=http%3A%2F%2Fjournals.plos.org%2Fplosone%2Fs%2Fsubmission-guidelines%23loc-laboratory-protocols&data=02%7C01%7C%7C3ed52e2abdaa49086d1e08d726e6f9e0%7C1faf88fea9984c5b93c9210a11d9a5c2%7C0%7C1%7C637020645783299346&sdata=Smehh%2FQQwYfM6iCAx29BuZ4vbNeHhoTYj4aRIwoQAP0%3D&reserved=0)

Please include the following items when submitting your revised manuscript:

- A rebuttal letter that responds to each point raised by the academic editor and reviewer(s). This letter should be uploaded as separate file and labeled 'Response to Reviewers'.
- A marked-up copy of your manuscript that highlights changes made to the original version. This file should be uploaded as separate file and labeled 'Revised Manuscript with Track Changes'.
- An unmarked version of your revised paper without tracked changes. This file should be uploaded as separate file and labeled 'Manuscript'.

Please note while forming your response, if your article is accepted, you may have the opportunity to make the peer review history publicly available. The record will include editor decision letters (with reviews) and your responses to reviewer comments. If eligible, we will contact you to opt in or out.

We look forward to receiving your revised manuscript.

Kind regards,

Yuankai Huo, Ph.D.
Academic Editor
PLOS ONE

**Journal Requirements**

1. When submitting your revision, we need you to address these additional requirements.

Please ensure that your manuscript meets PLOS ONE's style requirements, including those for file naming. The PLOS ONE style templates can be found at
<http://www.journals.plos.org/plosone/s/file?id=wjVg/PLOSOne_formatting_sample_main_body.pdf> and <http://www.journals.plos.org/plosone/s/file?id=ba62/PLOSOne_formatting_sample_title_authors_affiliations.pdf>

**All efforts have been made to ensure the manuscript meets PLOS ONE’s style requirements (change of level 1 and level 2 heading size, only first letter capitalized; tables included directly after the paragraph in which they are first cited; title page formatting change).**

2. Thank you for stating the following in the Competing Interests section:

"NCF’s research group has received payment for consultancy or for conducting studies from Biogen, Eli Lilly Research Laboratories, GE Healthcare, and Roche. NCF receives no personal compensation for the activities mentioned above. JMS has received research funding from Avid Radiopharmaceuticals (a wholly owned subsidiary of Eli Lilly), has consulted for Roche Pharmaceuticals, Biogen, Merck and Eli Lilly, given educational lectures sponsored by GE Healthcare, Eli Lilly and Biogen, and serves on a Data Safety Monitoring Committee for Axon Neuroscience SE."

Please confirm that this does not alter your adherence to all PLOS ONE policies on sharing data and materials, by including the following statement: "This does not alter our adherence to  PLOS ONE policies on sharing data and materials.” (as detailed online in our guide for authors <http://journals.plos.org/plosone/s/competing-interests>).  If there are restrictions on sharing of data and/or materials, please state these. Please note that we cannot proceed with consideration of your article until this information has been declared.

Please include your updated Competing Interests statement in your cover letter; we will change the online submission form on your behalf.

**We have updated the competing interests statement and included it in the cover letter as requested.**

**Comments to the Author**

1. Is the manuscript technically sound, and do the data support the conclusions?

The manuscript must describe a technically sound piece of scientific research with data that supports the conclusions. Experiments must have been conducted rigorously, with appropriate controls, replication, and sample sizes. The conclusions must be drawn appropriately based on the data presented. 

Reviewer #1: Yes

Reviewer #2: Yes

2. Has the statistical analysis been performed appropriately and rigorously? 

Reviewer #1: Yes

Reviewer #2: I Don't Know

3. Have the authors made all data underlying the findings in their manuscript fully available?

The [PLOS Data policy](https://eur01.safelinks.protection.outlook.com/?url=http%3A%2F%2Fwww.plosone.org%2Fstatic%2Fpolicies.action%23sharing&data=02%7C01%7C%7C3ed52e2abdaa49086d1e08d726e6f9e0%7C1faf88fea9984c5b93c9210a11d9a5c2%7C0%7C1%7C637020645783299346&sdata=kkSjRjnVt2rpDAswtYPp058di8Hn5sKOJklb7CqZLu4%3D&reserved=0) requires authors to make all data underlying the findings described in their manuscript fully available without restriction, with rare exception (please refer to the Data Availability Statement in the manuscript PDF file). The data should be provided as part of the manuscript or its supporting information, or deposited to a public repository. For example, in addition to summary statistics, the data points behind means, medians and variance measures should be available. If there are restrictions on publicly sharing data—e.g. participant privacy or use of data from a third party—those must be specified.

Reviewer #1: Yes

Reviewer #2: Yes

4. Is the manuscript presented in an intelligible fashion and written in standard English?

PLOS ONE does not copyedit accepted manuscripts, so the language in submitted articles must be clear, correct, and unambiguous. Any typographical or grammatical errors should be corrected at revision, so please note any specific errors here.

Reviewer #1: Yes

Reviewer #2: Yes

5. Review Comments to the Author

Please use the space provided to explain your answers to the questions above. You may also include additional comments for the author, including concerns about dual publication, research ethics, or publication ethics. (Please upload your review as an attachment if it exceeds 20,000 characters)

Reviewer #1: This is a very useful study that evaluated hippocampal subfields among putatively cognitively normal individuals with and without high and low amyloid low. The importance of this. Study lies in the delineation of hippocampal subfields that may be important in understanding early AD pathophysiology. Compared to β-amyloid negative participants (n=334), β-amyloid positive participants (n=74) had lower volume of the presubiculum (3.4% smaller, p=0.012).
This finding with the presubiculum and the fact that this region was not subjected to age effects. like other hippocampal subfields is intriguing.
Minor comments: A better description of specific white matter or other regions for SUVR normalization would be helpful.

**A more detailed description of the PET analysis pipeline has been included to address this and reviewer 2’s helpful comments:**

**“Concurrent acquisition of PET and MRI was performed on the same Siemens Biograph mMR 3 Tesla PET/MRI scanner. Static PET images representing uptake during a 10-minute period approximately 50 minutes after injection of approximately 370 megabecquerels of 18F-florbetapir were reconstructed using a thorughly validated pseudo-CT attenuation correction method (40). The post-uptake images were then rigidly registered to the structural MRI data using a symmetric block matching technique (41). A previously defined cortical grey matter composite (composed of frontal, temporal, parietal, and cingulate regions (42–46)) was selected as the primary region of interest to assess β-amyloid burden. All voxels in the image were then normalised to a reference region to produce a Standard Uptake Value Ratio (SUVR) image. The reference region selected was a mask of subcortical white matter, eroded one time to avoid partial volume effects. The advantages of the subcortical white matter as a reference region are that it is a relatively uniform tissue type and has less risk of corruption from other tissues compared to other commonly used reference regions (e.g. the cerebellum). Furthermore, SUVR values derived from a white matter reference region have also been shown to correlate better with gold standard arterial sampling based dynamic measurements of tracer uptake compared to SUVR values derived using other widely used reference regions (e.g. the cerebellum)(47). Gaussian mixture models were used to fit the data and obtain a threshold for β -amyloid positivity. Mixture models with one, two, and three gaussians, were tested, with the best model selected using Bayesian Information Criteria. The best fit for the composite cortical grey matter region of interest was two gaussians. The 99th percentile SUVR value of the Gaussian representing the β-amyloid negative population was selected as the cut-point (0.6104) for β-amyloid positivity”**

Ruling out a formal diagnosis of MCI by clinical evaluation and psychometric tests allows many persons into the study who presumably had neuropsychological impairment without subjective complaints and visa-versa. As such, it not clear whether these participants were actually “cognitively normal” and a number of studies have shown that persons diagnosed with PreMCI, not MC/I not cognitively normal (NACC) or even subjective cognitive complaints may have biological underpinnings. This should. be acknowledged and the authors may even want to run some correlation between the subiculum and other diagnostic tests. The face-name association test might be one such candidate since it was not used in diagnostic formulation.

**We thank the reviewer for these suggestions. We have as suggested run further analyses exploring the relationship between presubiculum volume and face-name association test, confirming there was no relationship. This has been included in the results:**

**“To explore whether the association observed between β-amyloid and presubiculum volume was driven by cognitively impaired individuals not captured by the MCI diagnostic criteria used to characterise the sample, a post-hoc regression analysis between presubiculum volume and the 12-item Face-Name test (i.e. a test of episodic memory not used in the diagnostic formulation in this study) was performed. There was no evidence that 12-item Face-Name test performance predicted presubiculum volume (p=0.82), nor was there evidence of an interaction between 12-item Face-Name test performance and β-amyloid status in terms of its effect on presubiculum volume (p=0.30).”**

While I am personally convinced that these are genuine and important findings, multiple tests of statistical significance were run without any reference for the potential of family-wise alpha error or false discovery rate.

**In order to acknowledge this point, we have included the following statement in the discussion:**

**“In addition, formal correction for multiple comparisons was not performed in this analysis, although efforts were made to constrain the analysis to total subfield volumes (sum of left and right) in the first instance and excluded subfields that were likely to be unreliable. Furthermore, to what extent hippocampal subfields are truly independent, and therefore to what extent it is valid to apply such techniques is unclear (16).”**

Overall, I really liked this paper and thought that it was well argued and well written.

Reviewer #2: The study provided evidence of differential associations in cognitively normal older adults between hippocampal subfield volumes and β-amyloid deposition and, increasing age at time of scan. The relatively selective effect of lower presubiculum volume in the β-amyloid positive group potentially suggest that the presubiculum may be an area of early and relatively specific volume loss in the pathophysiological continuum of Alzheimer’s disease.

Although I do not have any practical knowledge of the performed PET imaging analysis, I believe that the authors did not provide a well-structured, detailed and comprehensive description of the conducted analyses.

**We thank the reviewer for this comment – please see our response to reviewer 1 for an expanded version of the PET imaging analysis pipeline**

Also in the introduction and the discussion, the authors did not provided a comprensive overview of the most relevant literature to the presented work.

**As requested, we have expanded the introduction to provide more details of the literature:**

**“In a small sample (n=74), Hsu and colleagues reported Aβ-associated decreases in not only total hippocampal volume, but also the subiculum and pre-subiculum(26). The segmentation algorithm utilized in this study has been shown to be vulnerable to mislabelling(27) and there is a requirement for studies with larger sample sizes and more up to date hippocampal subfield segmentation methodology to further investigate this relationship.”**

**“Research into hippocampal subfield volumes in pre-clinical populations previously has been limited. Hsu and colleagues reported Aβ-associated decreases in the subiculum and pre-subiculum in a small sample of cognitively normal individuals (n=74)(26), the pattern of which is broadly consistent with the findings presented in this analysis. However, the magnitude of these volume differences was much greater (approximately 10-12% compared to 3-4% in Insight 46). A number of factors including smaller sample size and wide age range (with a trend for the Aβ-positive to be older) as well recruitment via convenience sampling in the previous study may account for these discrepancies. Furthermore, Hsu and colleagues utilized a previous version of Freesurfer’s hippocampal subfield segmentation algorithm, which has been shown to be vulnerable to mislabelling(27).”**

**“A recent study of 88 individuals with a family history of AD investigating hippocampal sub-region volumes found those with abnormal CSF Aβ, but normal CSF tau had increased right subiculum volumes, whilst abnormal CSF Aβ and abnormal CSF tau was associated with decreased right subiculum volume(21) suggesting the presence of tau deposition is important for hippocampal atrophy and has selective effects on certain subfields.”**

Also, it is not clear to me QC of the T1 scans and segmentation

**The following was included in the original manuscript:**

**“All MRI data were preprocessed for gradwarp and image inhomogeneity and underwent a detailed quality control process to ensure adequate coverage and contrast and absence of motion artefact”**

**We have expanded this section:**

**“All MRI data were preprocessed for gradwarp and image inhomogeneity(48,49). Furthermore, all MRI data underwent a detailed quality control process by trained assessor to ensure there was adequate coverage and absence of motion artefact. T1 scans were also assessed for blurring, image wrap-around and contrast problems, and FLAIR for adequate CSF suppression (31).”**

**In addition, the following has been included in the segmentation methods:**

**“Visual inspection of each participant’s hippocampal subfield segmentation and corresponding T1-weighted structural MRI data was performed to ensure each segmentation conformed to the hippocampus and there were no clear and obvious errors. This was performed with the caveat that the that precise visualisation of the boundaries that define the distinct hippocampal subfields at the spatial resolution used in this study is challenging (50).”**

I might have missed this, but were these any figures for results?

**No figures were included in the original manuscript. We have now included scatter plots highlighting the cross-sectional association between age at time of scan and hippocampal subfield volumes.**

6. PLOS authors have the option to publish the peer review history of their article ([what does this mean?](https://eur01.safelinks.protection.outlook.com/?url=https%3A%2F%2Fjournals.plos.org%2Fplosone%2Fs%2Feditorial-and-peer-review-process%23loc-peer-review-history&data=02%7C01%7C%7C3ed52e2abdaa49086d1e08d726e6f9e0%7C1faf88fea9984c5b93c9210a11d9a5c2%7C0%7C1%7C637020645783309344&sdata=EziMD6vLt%2Fos40Af5NazmncTwT1w1KtwHf4iZst%2BE8Q%3D&reserved=0)). If published, this will include your full peer review and any attached files.


If you choose “no”, your identity will remain anonymous but your review may still be made public.


**Do you want your identity to be public for this peer review?** For information about this choice, including consent withdrawal, please see our [Privacy Policy](https://eur01.safelinks.protection.outlook.com/?url=https%3A%2F%2Fwww.plos.org%2Fprivacy-policy&data=02%7C01%7C%7C3ed52e2abdaa49086d1e08d726e6f9e0%7C1faf88fea9984c5b93c9210a11d9a5c2%7C0%7C1%7C637020645783309344&sdata=JRqrZ3CL%2BMkQkGVg5VabVDSEZv723Gvh%2Bu1kM1Hp940%3D&reserved=0).

Reviewer #1: No

Reviewer #2: No


[NOTE: If reviewer comments were submitted as an attachment file, they will be attached to this email and accessible via the submission site. Please log into your account, locate the manuscript record, and check for the action link "View Attachments". If this link does not appear, there are no attachment files to be viewed.]


While revising your submission, please upload your figure files to the Preflight Analysis and Conversion Engine (PACE) digital diagnostic tool, [https://pacev2.apexcovantage.com/](https://eur01.safelinks.protection.outlook.com/?url=https%3A%2F%2Fpacev2.apexcovantage.com%2F&data=02%7C01%7C%7C3ed52e2abdaa49086d1e08d726e6f9e0%7C1faf88fea9984c5b93c9210a11d9a5c2%7C0%7C1%7C637020645783319337&sdata=CJSQZdSbMhFS4GGtBIBy5Ry6SuXLcgvPPPfKAv4fcf4%3D&reserved=0). PACE helps ensure that figures meet PLOS requirements. To use PACE, you must first register as a user. Registration is free. Then, login and navigate to the UPLOAD tab, where you will find detailed instructions on how to use the tool. If you encounter any issues or have any questions when using PACE, please email us at [figures@plos.org](mailto:figures@plos.org). Please note that Supporting Information files do not need this step.

*In compliance with data protection regulations, you may request that we remove your personal registration details at any time.* [*(Remove my information/details)*](https://eur01.safelinks.protection.outlook.com/?url=https%3A%2F%2Fwww.editorialmanager.com%2Fpone%2Flogin.asp%3Fa%3Dr&data=02%7C01%7C%7C3ed52e2abdaa49086d1e08d726e6f9e0%7C1faf88fea9984c5b93c9210a11d9a5c2%7C0%7C1%7C637020645783319337&sdata=FFsvrUD1kI1zxT%2FyxgQRaeA40Vo3xk1QYQBE4SRXpO4%3D&reserved=0)*. Please contact the publication office if you have any questions.*
